# The mammalian PYHIN gene family: Phylogeny, evolution and expression

**DOI:** 10.1186/1471-2148-12-140

**Published:** 2012-08-07

**Authors:** Jasmyn A Cridland, Eva Z Curley, Michelle N Wykes, Kate Schroder, Matthew J Sweet, Tara L Roberts, Mark A Ragan, Karin S Kassahn, Katryn J Stacey

**Affiliations:** 1 The University of Queensland, School of Chemistry and Molecular Biosciences, Brisbane, Qld, 4072, Australia; 2 Queensland Institute of Medical Research, 300 Herston Road, Brisbane, Qld, 4006, Australia; 3 The University of Queensland, Brisbane, Qld, 4072, Australia; 4 The University of Queensland, ARC Centre of Excellence in Bioinformatics, Brisbane, Qld, 4072, Australia; 5 The University of Queensland, Centre for Medical Genomics, Brisbane, Qld, 4072, Australia

**Keywords:** PYHIN, HIN-200, cytosolic DNA, ALR, IFI16, AIM2

## Abstract

**Background:**

Proteins of the mammalian PYHIN (IFI200/HIN-200) family are involved in defence against infection through recognition of foreign DNA. The family member absent in melanoma 2 (AIM2) binds cytosolic DNA via its HIN domain and initiates inflammasome formation via its pyrin domain. *AIM2* lies within a cluster of related genes, many of which are uncharacterised in mouse. To better understand the evolution, orthology and function of these genes, we have documented the range of *PYHIN* genes present in representative mammalian species, and undertaken phylogenetic and expression analyses.

**Results:**

No *PYHIN* genes are evident in non-mammals or monotremes, with a single member found in each of three marsupial genomes. Placental mammals show variable family expansions, from one gene in cow to four in human and 14 in mouse. A single HIN domain appears to have evolved in the common ancestor of marsupials and placental mammals, and duplicated to give rise to three distinct forms (HIN-A, -B and -C) in the placental mammal ancestor. Phylogenetic analyses showed that *AIM2* HIN-C and pyrin domains clearly diverge from the rest of the family, and it is the only PYHIN protein with orthology across many species. Interestingly, although AIM2 is important in defence against some bacteria and viruses in mice, *AIM2* is a pseudogene in cow, sheep, llama, dolphin, dog and elephant. The other 13 mouse genes have arisen by duplication and rearrangement within the lineage, which has allowed some diversification in expression patterns.

**Conclusions:**

The role of AIM2 in forming the inflammasome is relatively well understood, but molecular interactions of other PYHIN proteins involved in defence against foreign DNA remain to be defined. The non-AIM2 PYHIN protein sequences are very distinct from AIM2, suggesting they vary in effector mechanism in response to foreign DNA, and may bind different DNA structures. The *PYHIN* family has highly varied gene composition between mammalian species due to lineage-specific duplication and loss, which probably indicates different adaptations for fighting infectious disease. Non-genomic DNA can indicate infection, or a mutagenic threat. We hypothesise that defence of the genome against endogenous retroelements has been an additional evolutionary driver for PYHIN proteins.

## Background

Members of the PYHIN protein family have recently come to prominence as receptors mediating the detection of foreign DNA and initiating innate immune responses. Absent in melanoma 2 (AIM2) binds DNA in the cytosol of macrophages and mediates activation of the inflammasome pathway [1-4]. A second family member, p202, binds to cytosolic DNA and antagonises this pathway [4]. The inflammasome is a protein complex initiating activation of the protease precursor procaspase 1. Active caspase 1 cleaves proIL-1β and proIL-18 prior to their secretion as active inflammatory cytokines, and also leads to rapid lytic cell death termed “pyroptosis” [5]. AIM2-mediated responses are elicited by viruses such as mouse cytomegalovirus (MCMV) and vaccinia, the cytosolic bacteria *Francisella tularensis* and *Listeria monocytogenes*, and even extracellular bacteria such as *Streptococcus pneumoniae*[6-11]. AIM2 was necessary for effective control of *Francisella tularensis* and MCMV infection of mice [6,7,11].

Another PYHIN family member, human IFI16, was shown to mediate inflammasome responses to Kaposi’s sarcoma virus DNA in the nucleus [12]. Infection led to increased nuclear colocalisation of IFI16 with ASC, followed by emigration of both factors into the perinuclear region. IFI16 but not AIM2 knockdown decreased procaspase-1 cleavage in response to viral infection. On the other hand, IFI16 and mouse PYHIN protein p204 were found to play a role in the recognition of foreign DNA leading to induction of interferon-β (IFN-β), which is a pathway distinct from the inflammasome [13]. Induction of IFN-β by cytosolic DNA requires the adapter protein STING (stimulator of interferon genes), and subsequent activation of TANK-binding kinase 1 (TBK1) leading to phosphorylation and nuclear translocation of the transcription factor interferon regulatory factor-3 (IRF-3) [14-18]. IFI16 is not the only contender for such a role in DNA recognition, as recent work suggests that the unrelated helicase protein DDX41 is the DNA-binding protein primarily required for the early induction of signalling leading to IFN-β production whilst IFI16-mediated responses to DNA may prolong the induction of IFN-β later in the response [19]. Both IFI16 and DDX41 were reported to bind to STING [13,19]. Overall, study of the *PYHIN* gene family has been hampered by the complexity of the family in mouse, and a lack of understanding of orthology between mouse and human genes.

The *PYHIN* genes were identified as a cluster on mouse and human chromosome 1 and were named mouse *Ifi200* (“interferon inducible”) [20,21] and human *HIN-200* (“hematopoietic, interferon-inducible nuclear proteins with a 200 amino acid repeat”) [22]. They have more recently been annotated as the “*PYHIN*” family, acknowledging the defining features of an N-terminal pyrin domain and C-terminal HIN domain. There are four human PYHIN proteins: IFI16 (interferon inducible protein 16) [23], MNDA (myeloid nuclear differentiation antigen) [24], AIM2 [25] and IFIX (interferon inducible protein X) [26]. Publications have so far detailed seven mouse proteins p202(a/b), p203, p204, p205, p206, Aim2/p210, and Mndal (MNDA-like) as well as a number of predicted proteins [22,27-30]. Family members are predominantly nuclear proteins, some with defined nuclear localisation signals [22]. There is potential for regulated localisation, since acetylation of the nuclear localisation signal of IFI16 led to its cytosolic accumulation [31]. Some family members have characterised nuclear export sequences [32], suggesting they may shuttle in and out of the nucleus. In contrast, p202 and AIM2 lack nuclear localisation signals and reside in the cytoplasm of untreated cells [2,4], consistent with the role of AIM2 and p202 in the recognition of cytosolic DNA. p206 is also reported to have cytoplasmic location [27].

Prior to the finding that members of the family function in pathogen recognition, publications focused on roles in cell growth and cell cycle control, tumour suppression, apoptosis, DNA damage response, senescence, muscle and myeloid differentiation and autoimmunity [33-38]. These functions are comprehensively reviewed elsewhere [22,39-42]. There is as yet limited insight into the specific molecular roles of the proteins in these diverse functions. Various family members have been found to bind tumour suppressors such as p53, BRCA1 (breast cancer 1, early onset) and retinoblastoma protein [34,43-45], supportive of roles in cell cycle regulation, DNA repair and apoptosis. Interactions with a range of transcription factors and signalling molecules are also reported [46-50]. The novel roles being uncovered for PYHIN proteins in host defence now provide relevance for the long-established interferon-inducibility of these genes. Consistent with viral need to evade detection, several viral proteins are characterised to bind PYHIN family members [51-53].

The PYHIN proteins are defined by the possession of one or two 200-amino acid HIN domains at the C terminus, and a pyrin domain at the N terminus [22,40]. The roles of the HIN and pyrin domains are well established for AIM2-mediated inflammasome responses [1,2]. AIM2 recognises DNA via its HIN domain, and then recruits the inflammasome adapter protein ASC (apoptosis-associated speck-like protein containing a CARD) via homotypic interaction of pyrin domains. ASC itself recruits procaspase 1 via its caspase recruitment domain (CARD), resulting in intermolecular cleavage to give active caspase 1. The pyrin domain of AIM2 can therefore be considered the effector domain eliciting inflammasome formation. Pyrin domains (also known as PYD, PAAD or DAPIN) are also found in other proteins involved in inflammasome formation, such as NOD-like receptors. They are part of the death domain superfamily which also includes the death domain, death effector domain, and CARD [54]. Death domains form six-helix bundles and are frequently involved in recruitment of proteins in apoptotic and inflammatory responses through homotypic interactions.

The HIN domain is unique to the PYHIN family, and three distinct sequence classes, HIN-A, -B, and -C, have been defined [22]. The HIN domain was predicted to combine two oligonucleotide/oligosaccharide binding (OB)-folds [55]. OB-folds are five-stranded β-barrel structures found in a number of single stranded DNA (ssDNA)-binding proteins such as replication protein A and breast cancer 2, early onset (BRCA2). The OB-fold prediction is now supported by the crystal structures of the HIN domains of IFI16 and AIM2 [56,57]. The structure of double stranded (ds) DNA-bound proteins [56] showed that interaction between the HIN domains and DNA was primarily by electrostatic interaction with the sugar-phosphate backbone, explaining the DNA sequence-independent responses to cytosolic DNA. This work also suggested that in the absence of DNA, the pyrin domain is bound to the HIN domain in an autoinhibited state. Unterholzner *et al.* showed that the tandem HIN domains of IFI16 were more effective in DNA binding than its single HIN-B domain, with the HIN-A domain alone being ineffective [13]. Interestingly, recent work showed that IFI16 had a preference for binding cruciform structure DNA [58]. Native mouse p202, which also has two HIN domains, strictly bound to dsDNA and not ssDNA [4], and biological responses mediated via AIM2 are dependent on dsDNA, and are not elicited by ssDNA [4]. Beyond this, whether the HIN domains of different PYHIN family members have any specificity for particular DNA sequences or structures remains to be established.

The presence of four PYHIN family members in human has been known for a number of years, but the number of predicted mouse genes has increased with each new release of the mouse genome. In this paper, we describe the mouse, human and rat gene loci and proteins, address the issue of orthology between mouse and human genes and expression of the many mouse genes, and examine the evolution of the gene family within mammals. This provides a picture of a rapidly evolving locus with vastly different gene repertoires in different mammalian species and even within mouse strains. Phylogenetic analysis shows a clear distinction between AIM2 and other family members, suggesting divergence in function. Surprisingly, given the important role for AIM2 in host defence in mouse, *AIM2* appears only as a pseudogene in a number of different lineages, and appears to have been lost from genomes on several independent occasions during evolution.

## Results and Discussion

### The PYHIN Gene Locus in Mouse

**Figure 1:**
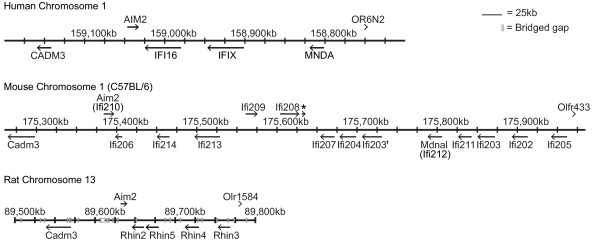
**Maps of the human, C57BL/6 mouse and rat* PYHIN *gene loci, based on NCBI human v36.3, mouse MGSCv37.2 and rat RGSCv3.4 assemblies.** The boundaries of the gene cluster in all three species are defined by the * CADM3 * gene and an olfactory receptor gene cluster. A number of bridged gaps are present in the rat locus, denoted by white boxes. The asterisk on the mouse locus denotes a HIN domain sequence, with expression supported by EST data. Accession numbers and alternative names for genes are provided in Table 1.

The *PYHIN* gene cluster is found in a syntenic region in many mammals, located between the *Cell Adhesion Molecule 3* (*CADM3)* gene and a collection of olfactory receptors and *spectrin alpha chain* (*SPTA1*), on chromosome 1q band H3 in mouse, 1q23 in human, and 13q24 in rat (Figure 1). On the basis of predicted genes and cDNA sequences, we PCR-cloned the open reading frames of 12 factors mapping to the locus from C57BL/6 splenic cDNA (*Ifi203-205, 207–214*) (Table 1, Figure 1) or cDNA from RAW264 cell line (*Ifi202*) and obtained cDNA for *Ifi206* from Ricky Johnstone (Peter MacCallum Cancer Centre, Melbourne). A fourteenth predicted gene [MGI: *Gm16340* can be discerned but there is only sparse EST evidence supporting its expression, compared with other genes. It encodes an intact open reading frame, and differs from *Ifi203* in only 25 amino acids out of 410, hence we have here termed it *Ifi203´*. The gene denoted here *Ifi207,* identified by Ludlow *et al.*[22] appears identical with *Ifi201*, for which a partial genomic DNA sequence had been published [21]. Although our cloned cDNA for *Ifi208* encodes a protein without a HIN domain, there is a HIN domain encoded by DNA immediately downstream of *Ifi208* (Figure 1), which is represented in EST databases. Thus a HIN-containing splice variant of *Ifi208* may exist.

**Table 1:** **Mouse, human and rat* PYHIN *gene accession numbers and nomenclature. Mouse sequences are from C57BL/6**

**Species**	**Name**	**Gene ID**	**Gene Symbol**	**Genbank Accession**	**Other Names**
Human	IFI16	3428	IFI16	NM_005531.2	PYHIN2
	IFIX	149628	PYHIN1	NM_198929.4	
	MNDA	4332	MNDA	NM_002432.1	PYHIN3
	AIM2	9447	AIM2	NM_004833.1	PYHIN4
Mouse	Ifi202	26388	Ifi202	NM_0119140.2	
	Ifi203	15950	Ifi203	NM_008328.2	
	Ifi203’	100504283	Gm16340	XM_003084464.1	
	Ifi204	15951	Ifi204	NM_008329.2	
	Ifi205	226695	Ifi205	NM_172648	D3', Ifi205a
	Ifi206	240921	Gm4955	XM_136331.8	
	Ifi207	226691	AI607873	NM_001204910.1	
	Ifi208	100033459	Pydc3	NM_001162938.1	
	Ifi209	236312	Pyhin1	NM_175026.2	Ifix
	Aim2/Ifi210	383619	Aim2	NM_001013779.2	
	Ifi211	381308	Mnda	NM_001033450.3	D3, Ifi205b, Pyhin3
	Mndal/Ifi212	100040462	Mndal	NM_001170853.1	
	Ifi213	623121	Pydc4	NM_001177349.1	
	Ifi214	-	-	JN200820.1	
Rat	Aim2	304987	Aim2	XM_222949.5	Rhin1
	Rhin2	289245	RGD1562462	XR_086006.1	
	Rhin3	304988	Ifi204	NP_001012029.1	Mnda, rHin-3
	Rhin4	498288	Ifi203-ps1	XR_086004.1	Ifi203
	Rhin5	689152	LOC689152	XM_002728037.1	

### Divergence of the locus between mouse strains

The *PYHIN* gene locus is divergent in different mouse strains. The region encoding *Mndal/Ifi212* is absent in a number of related mouse strains including DBA/2J, AKR/N, and NZB/BINJ [28]. Part of *Ifi203* is also missing in DBA [28], explaining the lack of *Ifi203* mRNA in that strain [59]. Zhang *et al.*[28] presented an arrangement of genes in the locus generated from two overlapping BAC sequences, one from C57BL/6 and one from 129X1/SvJ. This presents a model not consistent with the current C57BL/6 genome, and suggests that the 129 strain may be missing the genes *Ifi211* and *Ifi203* which lie between *Mndal/Ifi212* and *Ifi202* in C57BL/6 (Figure 1). In contradiction to this, an earlier map of the 129 strain mouse locus showed two extra duplications of *Ifi203*[20], one of which may be *Ifi203´ (Gm16340)*. In addition, there are two *Ifi202* genes and a pseudogene in the 129 mouse genome, designated *Ifi202ab* and *c*[20,60], but only one *Ifi202* gene in C57BL/6 (Figure 1), which has a number of minor sequence variations from the published *Ifi202a* and *b*[60]. Thus it seems that in the mouse lineage, the *PYHIN* locus has been subject to frequent amplification and rearrangement.

### Human and rat PYHIN loci

The human locus is simpler, with 4 identified genes (*AIM2, IFI16, IFIX, MNDA*, Figure 1). The human *AIM2* gene, like mouse *Aim2*, lies in the reverse orientation to other genes at one end of the gene cluster near the *CADM3* gene. Interestingly, the human locus may also be prone to duplications, as a recent paper reports copy-number variation, including within the *IFI16* gene, in the human population [61]. The rat genome is incomplete, with gaps remaining within the locus. There are currently four identifiable genes with both pyrin and HIN domains (*Aim2* and *Rhin2-4*) and an isolated pyrin domain (*Rhin5*) (Figure 1). EST databases support the expression of all the rat genes except *Rhin5*.

### Gene nomenclature

*PYHIN* gene and protein nomenclature has been confused over the years due to the similarity between genes. Early papers on a family member termed D3 [62] show sequence from the *Ifi211* gene, although this name also seems to have been used for the *Ifi205* gene, which is very similar. What we have termed *Ifi211* here has been annotated as *Ifi205b* in a recent review [30]. However, the N-terminal 200 amino acids encoded by *Ifi211* are more closely related to Ifi204 (98% amino acid identity), perhaps indicating recent gene conversion (Additional file 1: Figure S1). Consequently, we favour independent names for these genes. New names for genes have also been introduced during genome annotation on the basis of some inter-species sequence similarities. We show later in this paper that apart from *AIM2* there are no direct orthologues between mouse and human. Thus, we propose the simple numbering for mouse genes indicated in Table 1.

### The mouse, human and rat protein families

**Figure 2:**
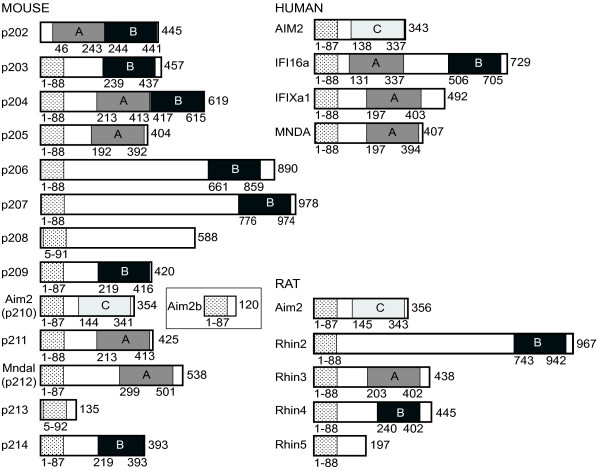
**Predicted protein domain organisation derived from cDNA sequences for human and mouse and predicted genes for rat.** Pyrin domains are indicated by stippled boxes, and the HIN domain subtypes (A, B or C) are shown. Only a single splice variant is shown for human IFI16 and IFIX, with complete details summarised previously [22]. Mouse p203 also has known splice variants [63]. Regions of sequence similarity among mouse proteins and the relationship between mouse and rat proteins are summarised in Additional Files 1 and 6.

The domain organisation inferred from cDNA sequences reveals that whilst most proteins have a pyrin domain at the N terminus and a C-terminal HIN domain, there are also cDNAs coding for proteins with no pyrin domain (p202), with two HIN domains (p202, p204, IFI16) and no HIN domains (p208, p213, AIM2b) (Figure 2). A number of splice variants exist for *IFI16, IFIX* and *Ifi203,* detailed elsewhere [22,63]. We have identified a splice variant of mouse *Aim2* that would generate a pyrin-only protein, termed here AIM2b (Genbank JQ894737).

### Identification of PYHIN genes in a range of species

Rigorous TBLASTN and iterative PSI-BLAST searches for PYHIN proteins in vertebrates revealed no trace of HIN domains outside of mammals, nor any other closely related domains. Although structural evidence suggests the HIN domain may have evolved from a DNA-binding OB-fold [55], there is now little primary sequence homology between HIN and other DNA-binding domains. PYHIN pyrin domains have some sequence similarity to pyrin domains found in NOD-like receptor proteins. Our searches in non-mammals using PYHIN pyrin domains returned only domains from these other protein families. TBLASTN searches in marsupials found only a single PYHIN coding sequence in each of opossum, Tasmanian devil and wallaby. Thus, at least one *PYHIN* gene existed prior to the divergence of marsupials and placental mammals. There is no evidence of any such gene in the platypus genome (NCBI Build 1.1, 6-fold coverage). Whether this truly reflects an absence in monotremes, or incomplete genome sequence, remains to be established*. PYHIN* gene sequences were identified in all major groups of mammals, except bats.

### Phylogenetic analysis supports HIN domain subtypes

To examine the evolution of the gene cluster during mammalian radiation, we used sequences from species in each of the major clades on the mammalian species tree (Table 1 and Additional File 2: Table S1). Evident shuffling of pyrin and HIN domain coding sequences between genes in mouse and in other species, as well as highly variable sequences in the region between pyrin and HIN domains meant that phylogenetic analyses of the protein sequences as a whole were not informative. Consequently, pyrin and HIN domains were used separately for analysis, with alignments of these domains provided in Additional File 3: Figure S3 and Additional File 4: Figure S4. Within the species shown, we extracted all the available complete (two-exon) HIN domain sequences.

**Figure 3:**
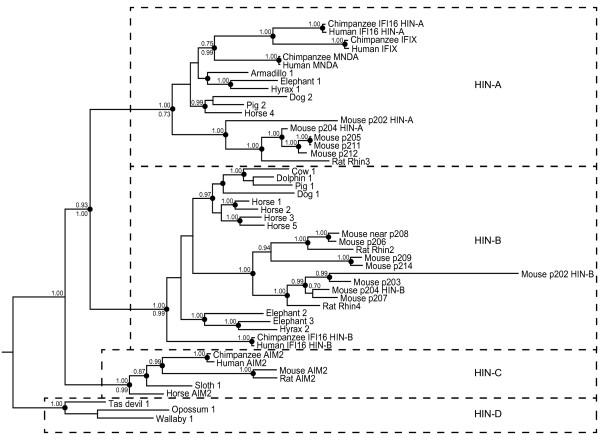
**Bayesian phylogenetic tree of HIN domains from a range of placental and marsupial mammals rooted with marsupial sequences.** Posterior probabilities ≥0.7 in this analysis are shown above the nodes. Clades that were supported by PhyML analysis (with likelihood-ratio test values >0.85, except as indicated) are shown by dots on the nodes. For a limited number of important nodes the PhyML likelihood-ratio test values are shown as a number below the node. Dotted lines separate observable HIN clades: placental HIN-A, -B, and -C, and marsupial HIN-D. Sequences used are specified in Table 1 and Additional File 2: Table S2, and the alignment is shown in Additional File 3: Figure S3.

Trees were generated by Bayesian as well as maximum likelihood approaches. A HIN domain phylogenetic tree derived from Bayesian analysis is shown in Figure 3, rooted with marsupial sequences. Overall, the tree topology was very similar by the two methods as indicated in Figure 3. This revealed three distinct clades of placental mammal sequences, consistent with previous assignment of HIN-A, HIN-B and HIN-C type domains [22]. The marsupial sequences cluster separately, and we have termed these HIN-D. The HIN-C domain is uniquely found in AIM2-like sequences, present at a maximum of one copy in each genome. A clear difference in the role of HIN-A, -B, and -C domains is yet to be established, although recombinantly expressed IFI16 HIN-A and HIN-B domains differ in their ability to bind DNA [13]. The maintenance of distinct HIN-A and HIN-B sequences amongst diverse species suggests that they may recognise different structures or sequences of DNA, and consequently be optimal for recognition of distinct classes of pathogens. Notable from this analysis is the branch length for mouse p202 HIN domains, particularly the HIN-B domain, indicating a rapid divergence from other mouse HIN domains.

### Pyrin domain phylogeny shows the distinct nature of AIM2

**Figure 4:**
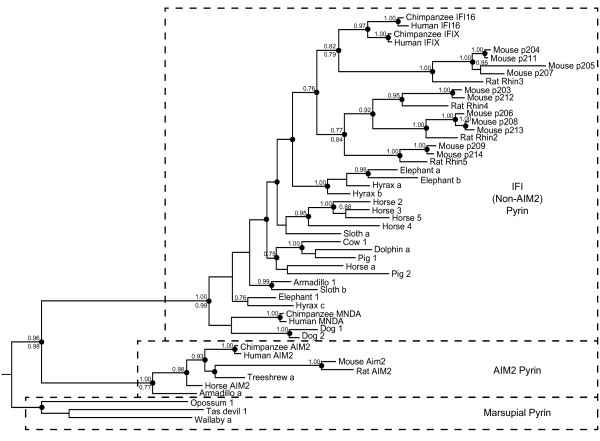
**Bayesian phylogenetic tree of PYHIN pyrin domains across placental and marsupial mammals, rooted with marsupial sequences.** Where a pyrin domain is clearly from the same gene as a HIN domain, labels are the same as Figure 3 (e.g. Cow 1). Some pyrin domains from low coverage genomes cannot definitively be linked to particular HIN domains, and are indicated with letters (e.g. Hyrax a). Posterior probabilities >0.7 are shown above nodes. Clades which were supported by PhyML analysis with likelihood-ratio test values >0.74 are shown by dots on the nodes. For a limited number of important nodes the PhyML likelihood-ratio test values are shown as a number below the node. Dotted lines indicate the three distinct clades: marsupial pyrin, pyrin domains from placental AIM2 sequences, and pyrin domains from placental non-AIM2 (IFI) sequences. Sequences used are defined in Table 1 and Additional File 2: Table S1, and alignment in Additional File 4: Figure S4.

Similar approaches were taken to generate phylogenetic trees for PYHIN pyrin domains, rooted with marsupial sequences. The resolution of these trees was limited by the short sequence of the pyrin domain (88 amino acids). However, the majority of clades were predicted similarly by Bayesian and maximum likelihood methods, as indicated in Figure 4. The major result is the demonstration of two markedly separate clades amongst the placental mammal proteins, one containing AIM2 orthologues, and another encompassing all other (“IFI”) PYHINs (Figure 4). The large divergence between the AIM2 and IFI sequences is evident by the long branch lengths separating these clades.

The maintenance of such a distinct pyrin domain in AIM2 suggests it has a different role from that of the other PYHIN proteins. Divergence of AIM2 and IFI pyrin domains occurred prior to placental mammal speciation, since diverse mammalian species have maintained distinct AIM2 and IFI pyrin sequences. In AIM2, the HIN domain binds DNA and the pyrin domain recruits ASC and nucleates the formation of the DNA inflammasome. Earlier results suggested that the AIM2 pyrin domain, but not other human PYHIN factors, could bind ASC [2]. Recent work suggests that IFI16 binds viral DNA in the nucleus and initiates inflammasome responses [12], although a direct interaction with ASC has not been demonstrated. Further work to examine proteins interacting with the IFI pyrin domains is warranted.

Amongst the non-AIM2 sequences, the pyrin tree topology (Figure 4) is markedly different from the HIN tree (Figure 3). There is no distinction between the pyrin domains of HIN-A and HIN-B containing proteins. In some cases, there has been evident gene conversion or domain shuffling which has maintained similarity of pyrin domain sequences within a species. For example, the two dog pyrin sequences are closely related, although one dog gene contains a HIN-A and one a HIN-B.

### Multiple species have AIM2 pseudogenes

**Figure 5:**
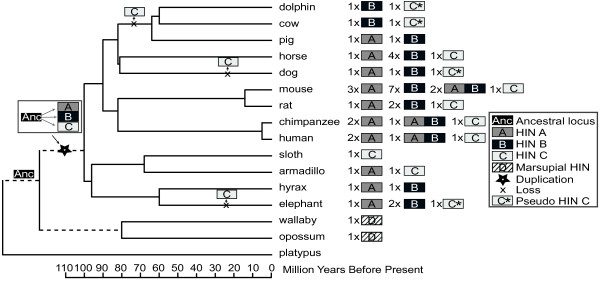
**Presence and absence of HIN domains in individual species, mapped onto a mammalian species tree**[64,67]. The number of HIN domains indicated must be considered a minimum estimate of the true number of HIN domains, particularly for the genomes with only 2-fold sequence coverage (dolphin, sloth, armadillo, hyrax and wallaby). The duplication of the ancestral gene to generate HIN-A, -B, and -C domains is indicated. Putative points of loss of a functional AIM2 HIN-C domain are marked, consistent with the presence of pseudogenes in a number of species, and likely absence of a gene in pig. Independent loss of AIM2 in dog as indicated, cannot be confirmed due to uncertainty in the phylogenetic relationship between horse, dog and Cetartiodactyla [65]. Armadillo has evidence for an *Aim2* HIN-C sequence, but as only one exon is available, this sequence was not used in phylogenetic analysis. Duplications and losses of HIN-A and HIN-B domains are numerous and are not indicated on the species branches here.

AIM2 is required for optimal defence against some viral and bacterial pathogens in mouse [6,7]. In contrast to the variable expansions of the other *PYHIN* family members, we have observed *AIM2* in only one copy per genome. Surprisingly, we found no intact *AIM2*, but evidence for an *AIM2* pseudogene in cow, llama, dolphin and sheep (Figure 5, Additional File 5: Figure S5) as well as elephant and dog (not shown). These sequences all encode HIN-C, more similar to AIM2 than other human IFI proteins (Additional File 5: Figure S5), but contain frame shifts and/or multiple stop codons. Cow, llama, dolphin and sheep are all part of the Cetartiodactyla [64], and there may have been basal loss of *AIM2* in the evolution of this clade (Figure 5). Pig also falls within this group, and we could find no discernible *AIM2* gene or pseudogene sequence within the available pig genome (Ensembl Sscrofa9), which has four-fold coverage. Horse appears to have an intact *AIM2* gene, whilst dog has a pseudogene. The phylogenetic relationship between horse, dog and the Cetartiodactyla is controversial [65], and an independent origin for the dog pseudogene, as shown in Figure 5, cannot be confirmed. However, generation of the elephant pseudogene must have been an independent event (Figure 5). It cannot be excluded that further sequencing may reveal intact *AIM2* in some of these species. However, the neighbouring *CADM3* gene can be reliably identified in the published genomes of all these species except sheep, which has relatively low coverage. Although AIM2 is of demonstrated importance in mouse [6,7], the probable loss of AIM2 in some species suggests they have evolved alternate mechanisms to cope with DNA viruses and intracellular bacteria.

### Lineage-specific gene family expansions

There are variable expansions of HIN-A and HIN-B encoding genes in different species, most prominently in mouse. Based on the phylogenetic analyses, clusters of mouse genes appear to have arisen by duplication after separation from the rat lineage (Figures 3 and 4). Between one and four mouse genes are co-orthologous to each of the five rat genes. *Rhin2* is orthologous to *Ifi206* (Figures 3 and 4) and is conserved in location in the locus adjacent to *Aim2* (Figure 1). *Rhin3* and *Rhin4* are orthologous to clusters of mouse HIN-A and HIN-B containing genes respectively. *Rhin5* is lacking a HIN domain coding sequence, but is otherwise orthologous to *Ifi214* and *Ifi209.* The relationship between mouse and rat proteins is summarised in Additional File 6: Figure S2. Apart from gene duplication in the mouse, there has been some domain swapping between paralogues and this provides a patchwork of sequence similarity between different proteins. For example, p212 is a hybrid between p203 and p205-like sequences [28,66]. The regions of sequence similarity between different mouse proteins are summarised in Additional File 1: Figure S1.

Lineage-specific duplications are also evident within horse, which has 6 recognisable genes, four of which form a clade on the pyrin tree (Figure 4). In contrast, the cow genome (UMD3.1 assembly) shows only a single intact *PYHIN* gene. The specific expansion of the gene family in some species may reflect the evolutionary “arms race” involved in combating infections. Gene duplication and diversification may allow better control of lineage-specific pathogens.

### Evolution of the PYHIN family

To describe the evolution of the PYHIN family, the HIN domain tree was reconciled with a mammalian species tree (Figure 5[64,67]). Duplication and deletion events were inferred based on the presence or absence of HIN domains and pseudogenes in different species. The model proposes that an ancestral HIN domain was amplified after the divergence of placental mammals and marsupials, and evolved into HIN-A, -B, and -C in the ancestor of all placental mammals (Figure 5). We found evidence for sequences for HIN-A, HIN-B and HIN-C domains within the Afrotheria (elephant and hyrax) and Xenarthra (armadillo and sloth), which are the most basal groups of placental mammals (Figures 3 and 5). The losses of the unique *AIM2* HIN-C domain described above are indicated in Figure 5. Some species show numerous pseudogenes containing HIN-A and HIN-B domains, but these are not considered here. The final number and composition of PYHIN proteins differs greatly between species, although dolphin, armadillo, sloth and hyrax genomes have been sequenced only at low coverage, and the genes indicated are likely a minimum estimate of the true number of genes in these species.

Branch lengths for both HIN and pyrin trees predict that AIM2 is somewhat closer in sequence to the *PYHIN* gene in the last common ancestor of placental mammals and marsupials, than are the other IFI sequences (Figures 3 and 4). This is most pronounced for the pyrin domain sequences (Figure 4). It is reasonable to propose that the ancestral function of the gene was similar to AIM2, in nucleating the inflammasome through recruitment of ASC. This function for the ancestral PYHIN would be expected, since ASC binding is a function also conserved in more distant pyrin domain-containing proteins such as NLRP3.

### Orthology between mouse and human

The identification of mouse and human orthologues is relevant to the use of mouse models for the study of human cellular processes and disease. Human and mouse AIM2 were the only direct orthologues identified between these species based on the phylogenetic analyses of both HIN and pyrin domains (Figures 3 and 4). The human IFI16 and IFIX proteins result from gene duplication within the primate lineage. The HIN-A domains of these proteins also cluster with higher probability with human and chimpanzee MNDA than with the clade of rodent HIN-A domains. This is consistent with speciation of rodent and primate lineages prior to amplification of the HIN-A domain. The C-terminal HIN domain in IFI16 is the sole human HIN-B domain. Rodent HIN-B domains are all more similar to one another than they are to the human. Thus there are no direct mouse orthologues of human IFI16, IFIX or MNDA. However, the pyrin domain tree shows that mouse p204, p211, p205 and p207 form a clade with human IFI16 and IFIX, rather than with other mouse proteins. If there is any functional distinction between the IFI pyrin domain clades, then these mouse proteins might have the closest effector function to IFI16 and IFIX.

Whilst it is tempting to infer common ancestry on the basis of shared arrangements of domains (i.e. IFI16 looks like p204), it is most likely that the acquisition of the double HIN domain structure in IFI16 and p204 arose by independent events, as there is no evidence for tandem HIN domains in any other non-primate species, including rat, and the phylogenetic analyses of HIN domain sequences did not support them as being orthologous. Despite the lack of 1:1 orthology between mouse and human in the non-AIM2 proteins, they may all be functionally quite similar. Gene duplications within a species could permit the evolution of different expression patterns or different intracellular locations, or may contain amino acid changes that circumvent the efforts of pathogens to evade detection. Gene expression patterns were investigated, to see whether the gene amplification had generated differential expression patterns.

### Expression of mouse genes

**Figure 6:**
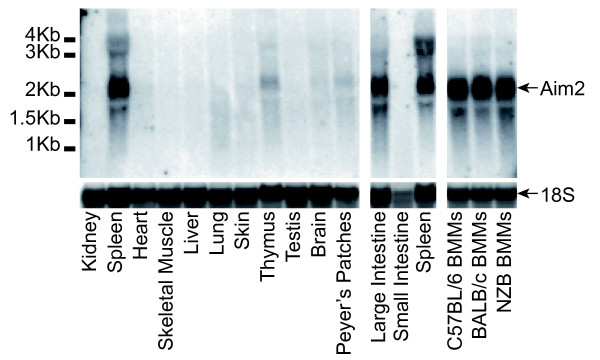
**Northern blots for*Aim2*expression in C57BL/6 mouse tissues, as well as bone marrow derived macrophages (BMM) from three mouse strains.** Results for 18 S rRNA are shown as a loading control. Tissues were perfused with saline to reduce blood cell contamination. Results are representative of two independent tissue samples.

**Figure 7:**
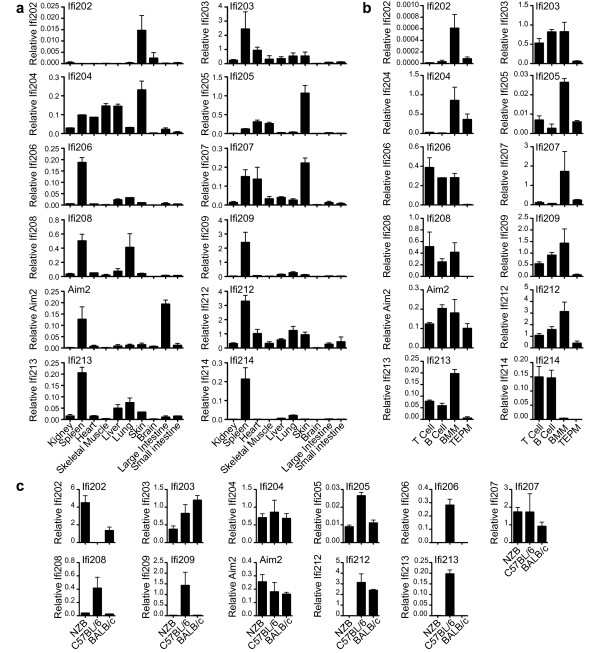
**Expression of mouse* PYHIN *genes in perfused C57BL/6 mouse tissues (a), in various C57BL/6 mouse immune cells – splenic T and B cells, BMM and thioglycollate elicited peritoneal macrophages (TEPM) (b), and in BMM from three different mouse strains (c).** Real time PCR results for each gene were normalised to the average of four housekeeping genes, which was found to give a relatively stable signal between tissues. Primers were tested to ensure lack of significant amplification of non-target *PYHIN* cDNAs. Results shown are the mean and range derived from duplicate RNA preparations.

Mouse AIM2 expression analysed by northern blot, showed predominant expression of AIM2 mRNA in spleen and large intestine (Figure 6). Real-time PCR generated a similar pattern of expression (Figure 7a). Previous work on human AIM2 found expression predominantly in spleen, with detectable mRNA in small intestine and peripheral blood but levels below detection in most other tissues tested [25]. Primers were designed for real-time PCR of all mouse family members, and tested for off-target amplification using cloned cDNAs from all mouse *PYHIN* genes. We were unable to design primers to specifically amplify *Ifi211*, due to its near identity to *Ifi204* at the 5´ end, and high similarity to *Ifi205204* and *212* in the rest of the gene. Screening of mouse tissues showed a variety of expression patterns. Spleen was a major site for most but not all factors. Tissues were obtained from C57BL/6 mice, which unlike other strains express negligible amounts of *Ifi202* mRNA in spleen [68]. However, C57BL/6 skin showed detectable *Ifi202* expression. *Ifi205204* and *207* showed strongest expression in skin, and interestingly these form a clade on the pyrin tree, together with *Ifi211* (Figure 4), for which we could not analyse expression. This group forms a larger clade with the pyrin domains of human IFIX and IFI16 (Figure 4). Apart from hematopoietic cell expression, IFI16 is expressed in epithelial cells and vascular endothelial cells [69-71]. It is prominently expressed in the lower proliferating layers of epithelia, including the skin [69]. Defence of proliferating keratinocytes against infection is a likely role for this group of skin-expressed human and mouse proteins.

*Ifi204* was also well expressed in liver, heart and skeletal muscle, and extensive work implicates p204 in muscle differentiation [39]. One paper has suggested that *Ifi203* expression at mRNA and protein level is limited to the liver [63], although we and others find it more widely expressed, with strongest expression in the spleen [28]. Overall, our results agree very well with those obtained for expression of *Ifi203-205* and *Mndal/Ifi212* in BALB/c mouse tissues by Zhang *et al.*[28]*,* and provide data for an extra seven genes.

*PYHIN* genes have been predominantly studied in hematopoietic cells, and here we have compared expression of the twelve genes in splenic T and B cells, as well as in bone marrow-derived macrophages (BMM) and thioglycollate-elicited peritoneal macrophages (Figure 7b). *Aim2* was readily detectable in all cells. Some genes such as *Ifi203, 206* and *213* were expressed well except in elicited macrophages. *Ifi204*, in contrast, was relatively myeloid restricted amongst the immune cell types, but is not restricted to myeloid cells since it had highest expression in skin (Figure 7a). The duplication of *PYHIN* genes allows for evolution of distinct patterns of regulation of similar genes. An example of this is seen with *Ifi209* and *Ifi214*, which result from recent duplication. Although on a tissue level they were similarly spleen-specific, on a cellular level *Ifi209* was expressed in T, B and BMM, whereas *Ifi214* was T and B cell-specific.

Lastly, we examined expression in BMM from three different mouse strains (Figure 7c). *Ifi202* showed marked differential expression, in agreement with previous work [4,68,72], and has been investigated as a possible susceptibility factor for lupus in the NZB mouse strain [41]. The lack of expression of *Mndal/Ifi212* in NZB is due to the lack of this gene in DBA and related strains [28]. There was no expression of *Ifi206Ifi209*, and *Ifi213*, and minimal expression detected for *Ifi208* in both NZB and BALB/c BMM. Interestingly, these genes are all clustered at one end of the gene array, near *Aim2* (Figure 1). This suggests that this region may be lacking in these strains. Strain-specific data are not shown for *Ifi214*, since this gene is barely expressed in BMM (Figure 7b).

The inter-strain differences complicate analysis of the *AIM2* gene trap knockout mice [6,7]. The gene trap allele is derived from 129 mouse ES cells and is generally used on a mixed 129 and C57BL/6 background. In the knockout mice, the *PYHIN* locus and chromosome 1 regions surrounding the null *AIM2* allele will derive only from 129 mice, whereas littermate controls with intact *AIM2* will carry the C57BL/6 gene array. Use of *AIM2* knockout mice on a pure background will thus be essential for many studies of AIM2 function in disease.

In summary, spleen and skin were the two most prominent sites of mouse *PYHIN* expression, consistent with a role in immune defence. Gene family expansion in mouse has allowed some diversification of expression patterns. Human and mouse *PYHIN* genes are variously expressed in a range of immune cell types and also epithelial and endothelial cells, although the genes do not obviously fill identical “niches”. For example MNDA is strongly myeloid restricted [73,74], but there seems to be no similarly expressed mouse gene. Apart from *AIM2*, the lack of concordance of expression patterns and sequence between mouse and human genes emphasises the need to consider the roles of individual proteins separately in the two species.

## Conclusions

Defences against invading DNA are likely to be found within all species, and are of course well characterised in the bacterial restriction/modification systems. The evolution of the first PYHIN protein is likely to have provided a new effector system for combating the threats indicated by stray DNA within mammalian cells. AIM2 appears less changed from the ancestral protein than are the other PYHIN proteins, and we anticipate the ancestral protein functioned similarly, in recruitment of the inflammasome in response to cytosolic DNA. A role for combating infectious threats is established for AIM2 [6,7], with both cytokine production and cell death likely to be important PYHIN-mediated defences. Duplication of the ancestral gene in the placental mammals may have enabled defence against organisms with a range of different replicative and evasion strategies. Since the HIN domain is the DNA-binding domain, the evolution of HIN-A, -B, and -C subtypes in placental mammals may have allowed specialisation to recognise different DNA structures. Differences in DNA-binding preferences of these domains are yet to be established. The pyrin domain evolution is constrained by maintenance of protein-protein interactions, such as recruitment of ASC for the AIM2 pyrin. Differences in interaction partners between AIM2 and the other PYHIN proteins warrants investigation, given the distinct natures of their pyrin domains. Further gene expansions prominent in mouse and horse may have provided particular advantage in combating species-specific infections. However, it is also possible that a driving force in evolution of the gene family is defence against high activity of endogenous retroelements, which create reverse transcribed DNA. Although evolution has been fuelled by waves of activity of retroelements [75], levels of activity need to be controlled to prevent excessive genome damage within each generation, and within somatic cells. Retroelements are generally silenced by methylation, and there is increasing evidence for their role in cancer and autoimmunity [76,77]. Cell death, as is mediated by AIM2 in response to cytosolic DNA, may be an appropriate response to eliminate cells where retroelements are inappropriately active. Interestingly, the estimated ages of retroelements in human and mouse genomes show ongoing high activity in the mouse genome but not human genome [78]. Thus it is conceivable that *PYHIN* expansion in the mouse could be driven by a need to control retroelement activity, as well as infectious disease.

## **Methods**

### cDNA cloning of mouse PYHIN family

To find novel PYHIN family coding sequences, DNA sequence based on the Pfam Pyrin domain was used to screen the NCBI Riken Transcripts Database. The NCBI databases and Ensembl genome browser also provided predicted genes in the *PYHIN* locus. Primers were designed to amplify complete coding sequences of published and novel mouse *PYHIN* genes, and PCR reactions were performed on cDNA from C57BL/6 mouse spleen and cloned into pEF6-TOPO-TA vector (Invitrogen).

### Genome searches for PYHIN family members

To assess the range of species in which PYHIN proteins could be discerned, TBLASTN searches were conducted against the GenBank whole-genome shotgun sequence database, using human AIM2 and IFI16 protein sequences and an expect threshold (E) of 10. From this a number of genomes spanning major placental mammal lineages, as well as all three available marsupial genes were selected for comprehensive analysis of *PYHIN* genes. Genomes analysed were: human (*Homo sapiens* NCBI v36.3), mouse (*Mus musculus* MGSCv37.2), rat (*Rattus norvegicus* RGSCv3.4), horse (*Equus caballus* EquCab2), dog (*Canis lupus familiaris* CanFam2.0), cow (*Bos taurus* UMD3.1), pig (*Sus scrofa* ENS:Sscrofa9), chimpanzee (*Pan troglodytes* CHIMP2.1.4), elephant (*Loxodonta Africana* loxAfr3), dolphin (*Tursiops truncates* turTru1), hyrax (*Procavia capensis* proCap1), sloth (*Choloepus hoffmanni* choHof1), armadillo (*Dasypus novemcinctus* dasNov2), opossum (*Monodelphis domestica* MonDom5), wallaby (*Macropus eugenii* Meug_1.0), and Tasmanian devil (*Sarcophilus harrisii* DEVIL7.0). Ensembl and NCBI genomic sequence databases were searched using TBLASTN and individual pyrin and HIN domain amino acid sequences from human AIM2 and IFI16, using default settings, and also with the gap opening penalty reduced to −9 and the expect threshold increased to 10. Genomes were also searched using a predicted protein from within the same genome to detect potential paralogs not detected by searching with human AIM2 or IFI16. The rat, opossum, dog and horse genomes were also searched using HIN and pyrin domain motifs using the MEME [79] and MAST v4.3.0 [80] software suite. Motifs that crossed different exons of the HIN domain were avoided. The motifs were used to search Ensembl *ab initio* predicted proteins. When novel unannotated genes were detected, genomic sequences containing PYHIN genes were examined for potential splice sites in conserved positions, and exons encoding a pyrin domain or the two exons encoding the HIN domain were extracted.

### Analysis of platypus and non-mammalian genomes

The platypus genome (*Ornithorhynchus anatinus* Build 1.1) was searched using TBLASTN for the full-length predicted PYHIN protein from opossum and full-length human IFI16. In another approach, all HIN domains identified in the 6x coverage mammalian genomes were used to build a profile hidden Markov model with the software HMMER [81], which was then used to screen the platypus *ab initio* peptide predictions available from Ensembl. An inferred ancestral HIN domain sequence from the node that predates the split between HIN-A, -B and -C domains was also used to perform this search. The genomes of chicken (*Gallus gallus* release 2.1), frog (*Xenopus tropicalis* version 4.1), anole lizard (*Anolis carolinensis* AnoCar1.0) and zebrafish (*Danio rerio* Zv8), available via NCBI and Ensembl, were searched using TBLASTN with the HIN domain from opossum, and human AIM2 and IFI16. None of these searches, nor the general search of all shotgun genomic sequences, yielded any trace of *PYHIN* genes in monotremes or non-mammals.

### Phylogenetic analyses

Phylogenetic analyses were performed separately on pyrin and HIN domains. The linking region between these domains was not used in the alignments as it is highly variable between individual proteins, and it was not possible to identify this region in the low coverage genomes. Alignments were constructed using ClustalW2 [82,83].

Phylogenetic inference was performed using Bayesian and maximum-likelihood approaches. For Bayesian analyses we used MrBayes v3.1.2 [84], running 2 million generations and 4 chains for each analysis, setting a burn-in of 2500 samples and sampling the tree space every 100 generations. Model jumping between fixed-rate amino acid models was used to determine the most-suitable substitution model, otherwise using default parameters. All analyses favoured the Jones substitution model [85]. Convergence was judged using the standard deviation of split frequencies and the plot of log likelihoods. Rooted trees were drawn using Mesquite v2.6 [86].

Maximum-likelihood analyses were performed using PhyML 3.0 [87] under the LG substitution model [88] with 4 substitution rate categories. The proportion of invariable sites and the gamma shape parameter were estimated from the data. We used 10 random starting trees, and subtree pruning and regrafting (SPR) [89] to search tree space. Both tree topology and branch lengths were optimized to maximize the likelihood. Branch support was estimated using 100 bootstrap replicates, and by calculating an approximate likelihood-ratio test [90].

### Isolation of mouse splenic immune cells

Mice were used as a source of tissues, under approval from the University of Queensland animal ethics committee (Approval number IMB/874/08). Splenocytes from C57BL/6 mice were harvested by homogenization of mouse spleens and red blood cells lysed by standard Gey’s solution procedure. CD19+ B cells and CD90.2+ T cells were isolated by positive selection using MACS microbeads, run according to manufacturer’s instructions (Miltenyi Biotec) over multiple columns. The purity of B and T cells assessed by flow cytometry was >98%. Bone marrow-derived macrophages from BALB/c, C57BL/6 or NZB mice were derived and cultured as described [91]. NZB mice were supplied by Kew animal house (Walter and Eliza Hall Institute, Australia), BALB/c by Animal Resources Centre, Perth, and C57BL/6 by University of Queensland Biological Resources. Thioglycollate elicited peritoneal macrophages were harvested from C57BL/6 mice and cultured as described [92].

### RNA isolation and real-time PCR

RNeasy Total RNA isolation kits (QIAGEN) were used to isolate RNA from mouse cell preparations, as well as from perfused C57BL/6 mouse tissues, according to standard protocols (QIAGEN). All tissues except bone marrow, spleen, liver, kidney and brain included a Proteinase K digestion step. cDNA was synthesised as previously described [93]. Where there are known splice variants of mouse PYHIN mRNAs, primers were designed to detect all variants (Additional File 7: Table S2). Efficiency of amplification was checked for the target gene. Specificity of each of the mouse PYHIN primer sets was assessed by running qPCR using a standard amount of each of the various different cloned PYHIN cDNAs as templates. Specificity was considered to be adequate when amplification of the target gene was achieved more than 10–12 cycles ahead of any other gene. Appropriate specificity was not achieved for *Ifi211*, and it could not be analysed here. Primers for *Ifi209* also amplified *Ifi214*. However, primers for *Ifi214* were specific and samples had a low level of *Ifi214,* which had minimal effect on the *Ifi209* expression data.

Levels of gene expression are shown for the test gene relative to the average expression of four house-keeping genes (*Tbp, Hprt, Cxxc1, Rpl13A*), performed in duplicate. These were quantified by RT PCR using the ΔCT method [94] as described [4]. Single exon primers were also tested against reactions containing no reverse transcriptase to ensure no genomic DNA contamination.

### Northern Blotting

10 μg of tissue RNAs and 7.5 μg of immune cell RNAs were resolved on a 1% MOPS/formaldehyde gel. The RNA was transferred to a Zeta-probe membrane (BioRad) by capillary blotting, and hybridised with AIM2 coding region cDNA probe at 65 °C or an oligonucleotide probe to 18 S rRNA at 42 °C according to manufacturer’s protocols (Biorad). The AIM2 probe was labelled as per the Amersham MegaPrime DNA Labelling System (GE Healthcare), and the 18 S rRNA probe was labelled using γ^32^P-ATP and polynucleotide kinase.

## Abbreviations

AIM2: Absent in melanoma 2; ASC: Apoptosis-associated speck-like protein containing a CARD; BMM: Bone marrow derived macrophages; BRCA1: Breast cancer 1, early onset; BRCA2: Breast cancer 2, early onset; CADM3: Cell adhesion molecule 3; CARD: Caspase recruitment domain; Cxxc1: CXXC Finger protein 1; DDX41: DEAD (Asp-Glu-Ala-Asp) box polypeptide 41; dsDNA: Double stranded DNA; HIN-200: Hematopoietic, interferon-inducible nuclear proteins with a 200 amino acid repeat; HPRT: Hypoxanthine guanine phosphoribosyl transferase; IFI200: Interferon inducible gene family; IFI16: Interferon inducible protein 16; IFIX: Interferon inducible protein X; IFN-β: Interferon- β; MCMV: Mouse cytomegalovirus; MNDA: Myeloid nuclear differentiation antigen; Mndal: MNDA-like; NLRP3: Nod-like receptor family, pyrin domain containing 3; OB-folds: Oligonucleotide/oligosaccharide binding folds; proIL-1β: Pro-interleukin-1β; proIL-18: Pro-interleukin-18; PYHIN: Pyrin and HIN domain-containing; Rhin: Rat HIN-200 family member; RPL13A: Ribosomal protein L13a; SPTA1: Spectrin alpha chain; ssDNA: Single stranded DNA; TBP: TATA box binding protein.

## Competing Interests

The authors have no competing interests to declare.

## Authors’ Contributions

JAD- cloning cDNAs and sequencing, bioinformatic searches, expression analysis, manuscript preparation, EZC- bioinformatic searches, expression analysis, MNW- purification of mouse B and T cells, KS- expression studies, MJS- mouse tissue cDNA, TLR- cloning mouse cDNAs, MAR- oversight of phylogenetic strategy, KSK- supervision of bioinformatic searches, phylogenetic analysis, manuscript preparation, KJS- project initiation and oversight, bioinformatic searches, manuscript preparation. All authors read and approved the final manuscript.

## Supplementary Material

Additional file 1 **Figure S1.** Diagram of subsequence composition of mouse PYHIN proteins. Click here for file

Additional file 2 **Table S1.** Sources of sequence data for PYHIN proteins from animals other than mouse, rat and human. Click here for file

Additional file 3 **Figure S3.** HIN domain alignment. Click here for file

Additional file 4 **Figure S4.** Pyrin domain alignment. Click here for file

Additional file 5 **Figure S5.** Evidence for cow, dolphin, llama and sheep AIM2 pseudogenes. Click here for file

Additional file 6 **Figure S2.** Comparison of mouse and rat PYHIN proteins. Click here for file

Additional file 7 **Table S2.** Sequences of primers for real time PCR. Click here for file
