# Supplementary material for: The mammalian PYHIN gene family: Phylogeny, evolution and expression
Source: BMC Evol Biol. 2012 Aug 7;12:140. doi: 10.1186/1471-2148-12-140 (PMC3458909; doi:10.1186/1471-2148-12-140)
Supplement: Additional file 1 — Figure S1. Diagram of subsequence composition of mouse PYHIN proteins. [file 1471-2148-12-140-S1.pdf]

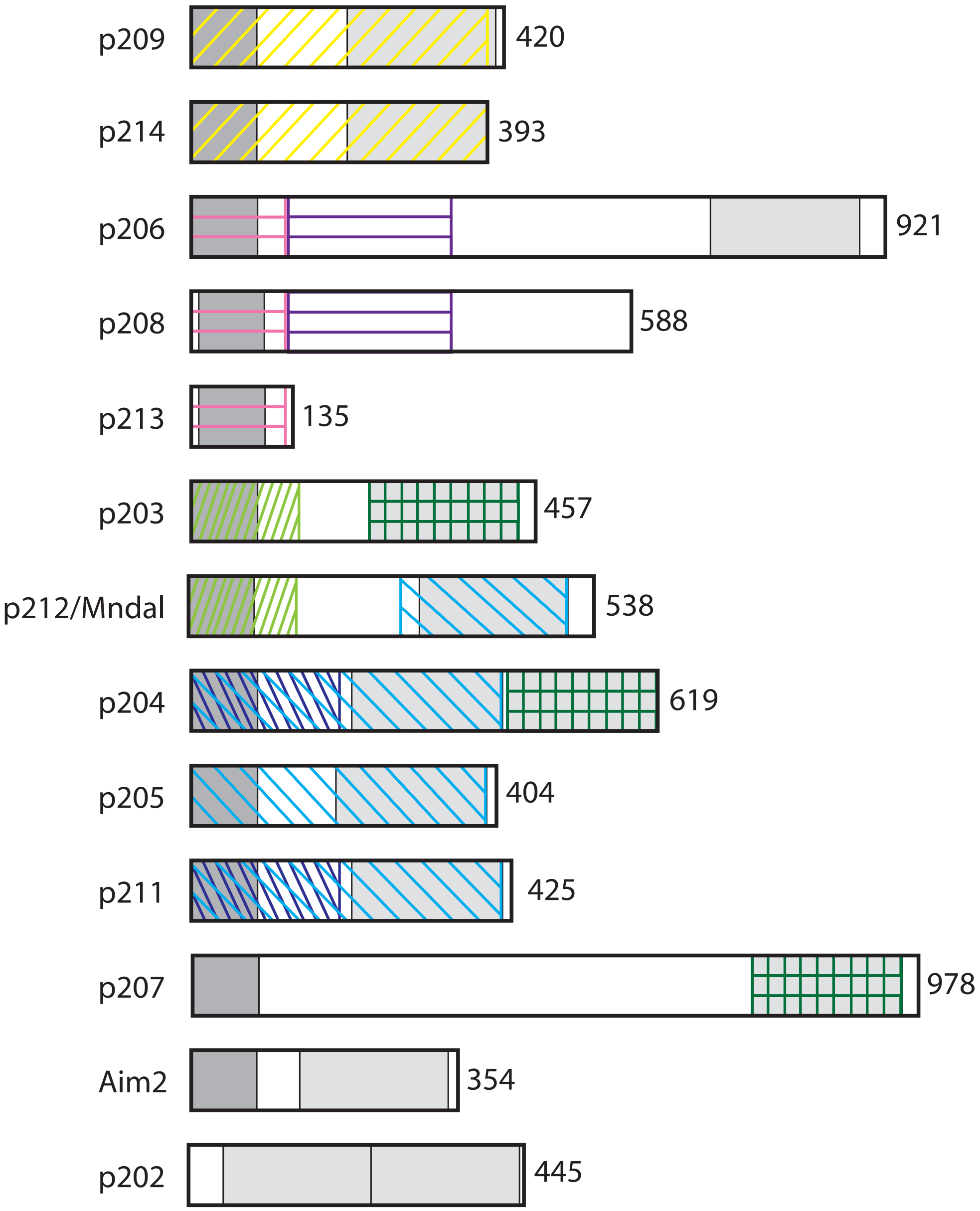

Supplementary Figure 1. Diagrammatic representation of sub-sequence composition of mouse PYHIN proteins. Gray shading indicates the location of pyrin and HIN domains. Coloured hatching shows regions of high sequence similarity (amino acid identity >80%). For example, p212 has an N terminal region showing 90.9% similarity with the starting region of p203 (light green hatching) and a C terminal region most similar to p204, p205 and p211 (light blue hatching), all of which are highly similar from the start of the gene to the end of the HIN-A domain. The dark blue hatching designates a region of particularly high identity between p211 and p204 (98%), which would be consistent with recent gene conversion. p202, although comprised of both HIN-A and HIN-B domains like p204, likely arose independently (see Figures 3 and 4), and so strong sequence similarity is not indicated.
