# Supplementary material for: The mammalian PYHIN gene family: Phylogeny, evolution and expression
Source: BMC Evol Biol. 2012 Aug 7;12:140. doi: 10.1186/1471-2148-12-140 (PMC3458909; doi:10.1186/1471-2148-12-140)
Supplement: Additional file 2 — Table S1. Sources of sequence data for PYHIN proteins from animals other than mouse, rat and human. [file 1471-2148-12-140-S2.pdf]

Supplementary Table 1. Summary of sources of sequence data for HIN and pyrin domains, for all animals other than human, mouse and rat, which are given in Table 1 of the manuscript.

Species	Accession Numbers	Pyrin Tree Label	HIN Tree Label
Armadillo	GenBank: AAGV020383493.1	Armadillo 1	Armadillo 1
	GenBank: AAGV020335327.1	Armadillo a	-
Chimpanzee	GenBank: XP_525227.2	Chimpanzee Ifix	Chimpanzee Ifix
	GenBank: XP_525225.2	Chimpanzee Mnda	Chimpanzee Mnda
	GenBank: XP_513914.2	Chimpanzee Aim2	Chimpanzee Aim2
	GenBank: XP_513913.2	Chimpanzee Ifi16	Chimpanzee Ifi16
Cow	GenBank: XP_869021.2	Cow 1	Cow 1
Dog	GenBank: XP_003434347.1	Dog 1	Dog 1
	GenBank: XP_545739.2	Dog 2	Dog 2
Dolphin	GenBank: ABRN01216818.1		Dolphin 1
	GenBank: ABRN01216816.1	Dolphin a	
Elephant	GenBank: NW_003573453.1	Elephant 1	Elephant 1
	GenBank: NW_003573453.1		Elephant 2
	GenBank: NW_003573453.1		Elephant 3
	GenBank: NW_003573453.1	Elephant a	
	GenBank: NW_003573453.1	Elephant b	
Horse	Ensembl Genscan prediction: GENSCAN00000027147		Horse 1
	Ensembl Genscan prediction: GENSCAN00000027123	Horse a	
	Ensembl Genscan prediction: GENSCAN00000027134	Horse 2	Horse 2
	GenBank: XR_035879.2	Horse 3	Horse 3
	GenBank: XP_001490553.2	Horse 4	Horse 4
	GenBank: XP_001490598.2	Horse 5	Horse 5
	GenBank: XP_001915358.1	Horse Aim2	Horse Aim2
Hyrax	GenBank: ABRQ01399281.1		Hyrax 1
	GenBank: ABRQ01467223.1		Hyrax 2
	GenBank: ABRQ01154641.1	Hyrax a	
	GenBank: ABRQ01441138.1	Hyrax b	
	GenBank: ABRQ01399280.1	Hyrax c	
Opossum	GenBank: NW_001581871.1	Opossum 1	Opossum 1
Pig	GenBank: XP_001929247.1	Pig 1	Pig 1
	GenBank: XP_003125734.1	Pig 2	Pig 2
Sloth	GenBank: ABVD01220630.1		Sloth 1
	GenBank: ABVD01534538.1	Sloth a	
	GenBank: ABVD01614599.1	Sloth b	
Tasmanian devil	GenBank: GL972687	Tas. devil 1	Tas. devil 1
Treeshrew	GenBank: AAPY01470609.1	Treeshrew a	
Wallaby	GenBank: ABQO010679940.1	Wallaby a	
	GenBank: ABQO010283775.1 GenBank: ABQO010284350.1		Wallaby 1
