# Supplementary figures and images for: The mammalian PYHIN gene family: Phylogeny, evolution and expression
Source: BMC Evol Biol. 2012 Aug 7;12:140. doi: 10.1186/1471-2148-12-140 (PMC3458909; doi:10.1186/1471-2148-12-140)

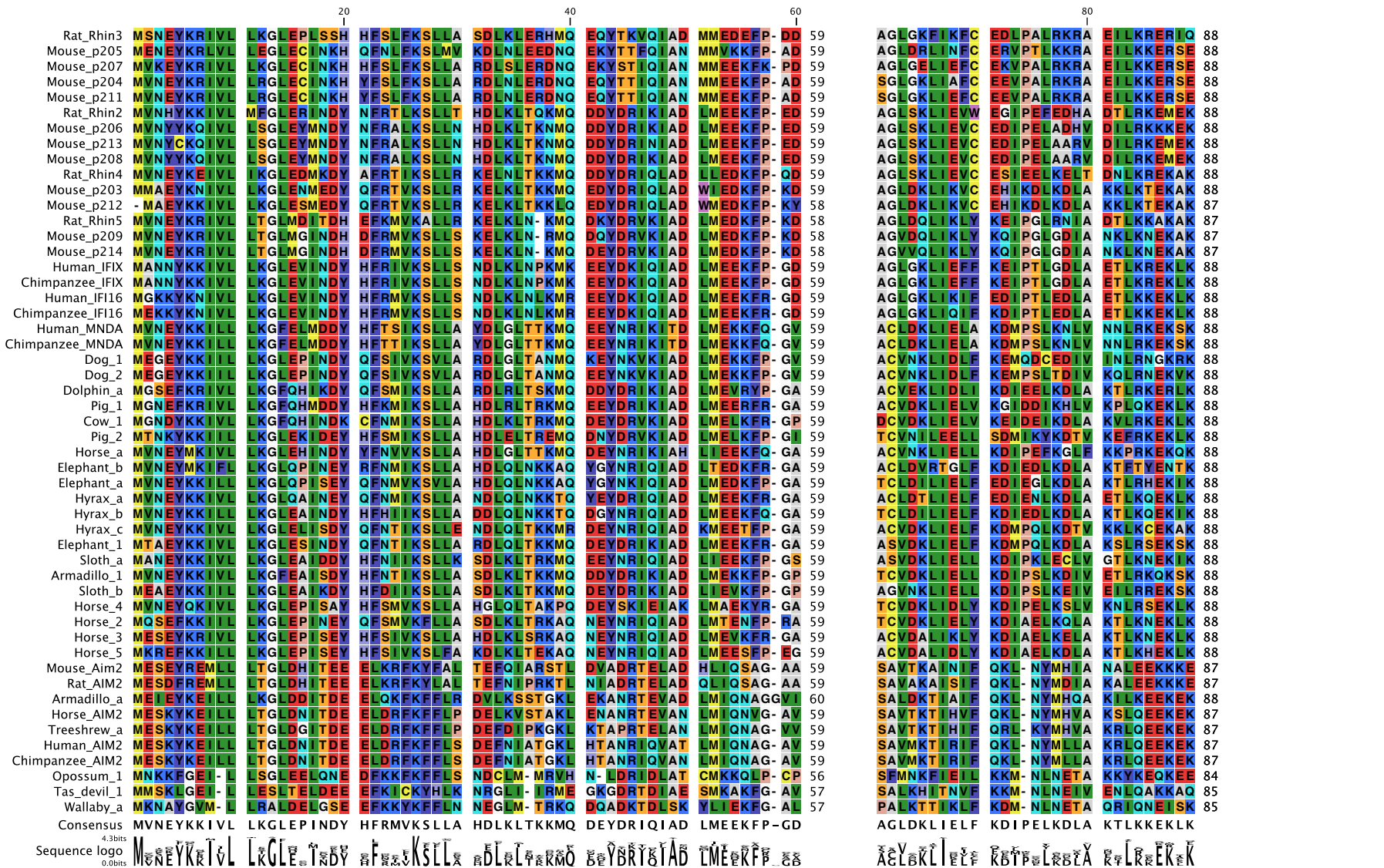

Supplement: Additional file 4 — Figure S4. Pyrin domain alignment. [file 1471-2148-12-140-S4.pdf]
