# Supplementary material for: The mammalian PYHIN gene family: Phylogeny, evolution and expression
Source: BMC Evol Biol. 2012 Aug 7;12:140. doi: 10.1186/1471-2148-12-140 (PMC3458909; doi:10.1186/1471-2148-12-140)
Supplement: Additional file 5 — Figure S5. Evidence for cow, dolphin, llama and sheep AIM2 pseudogenes. [file 1471-2148-12-140-S5.pdf]

a.

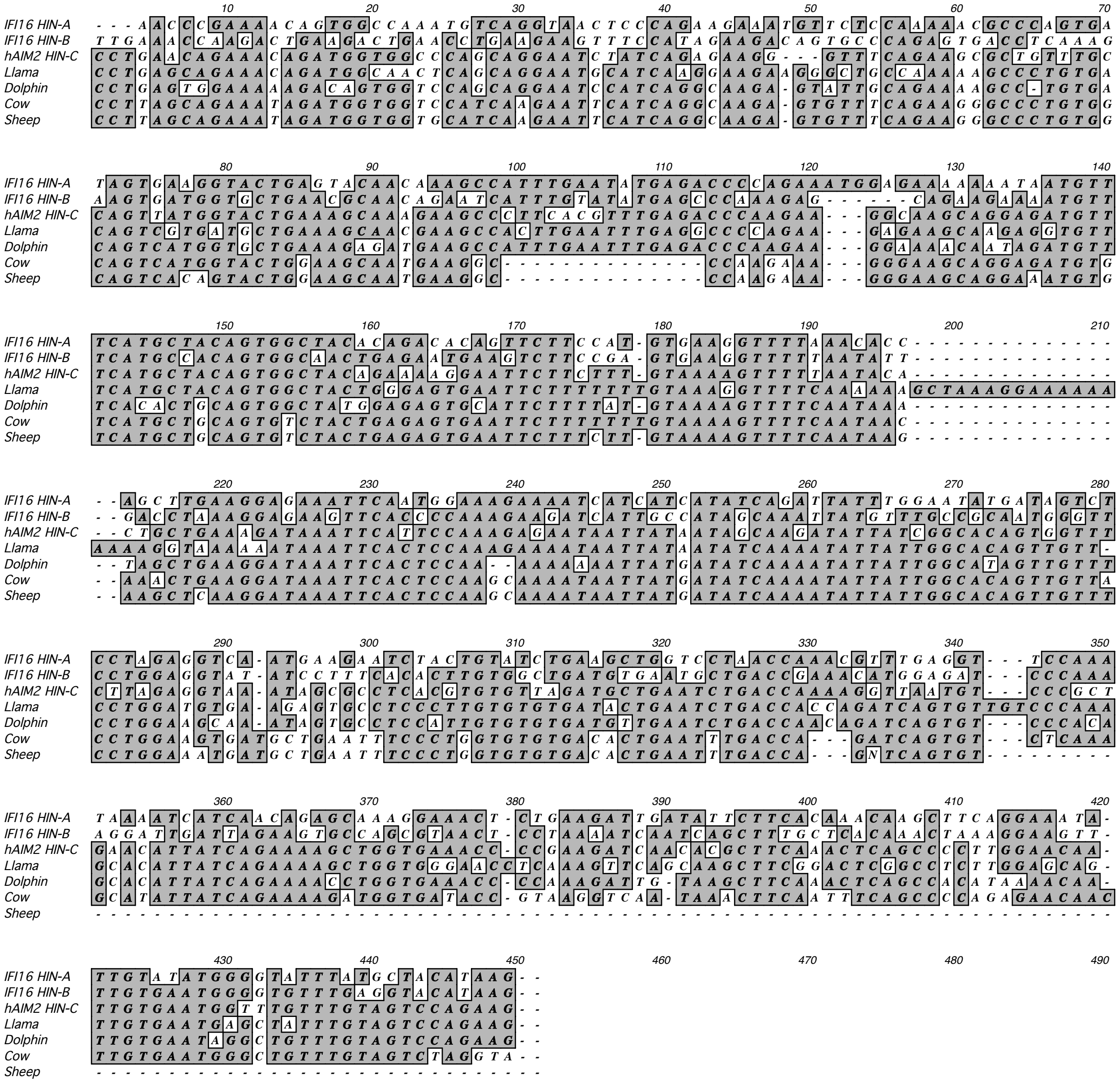

b.

	AIM2 HIN-C	IFI16 HIN-A	IFI16 HIN-B
cow	77	62	61
llama	80	73	72
dolphin	79	67	67

Supplementary Figure 5. BLAST searches of dolphin, cow, llama and sheep genome sequences with the AIM2 HIN domain revealed an *AIM2* pseudogene, and no trace of an intact *AIM2* gene. Sequences were obtained by TBLASTN searches on the dolphin, cow and lama genomes (Ensembl) using human AIM2 HIN-C domain, and a BLAST search on the sheep whole genome shot-gun sequence database (NCBI) using the cow pseudogene sequence. **a.** A clustalW alignment is shown between the first exon encoding human IFI16 HIN-A, IFI16 HIN-B, human AIM2 HIN-C, and sequences obtained from cow, dolphin, lama and sheep. Multiple frameshifts and/or stop codons are present in the cow, dolphin, lama and sheep sequences. **b.** Pairwise comparisons of these sequences shown in the table reveal that the pseudogenes obtained have higher similarity to DNA encoding the HIN-C domain of AIM2, than to HIN-A or HIN-B encoding DNA. Pig, which falls within the same evolutionary clade as the animals studied here was searched extensively for *AIM2*, and showed no gene or pseudogene with closer homology to *AIM2* than to *IFI16*. *AIM2* pseudogenes are also seen in dog and elephant (not shown).
