# Supplementary material for: The mammalian PYHIN gene family: Phylogeny, evolution and expression
Source: BMC Evol Biol. 2012 Aug 7;12:140. doi: 10.1186/1471-2148-12-140 (PMC3458909; doi:10.1186/1471-2148-12-140)
Supplement: Additional file 6 — Figure S2. Comparison of mouse and rat PYHIN proteins. [file 1471-2148-12-140-S6.pdf]

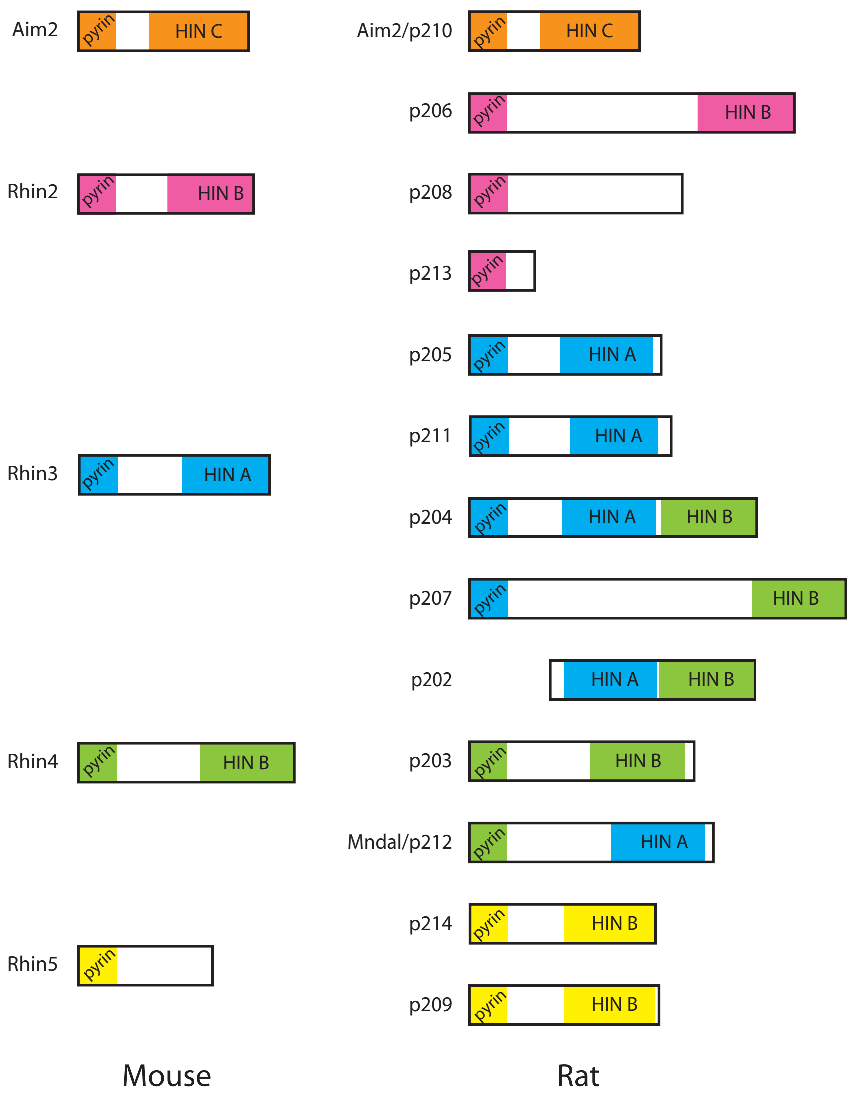

Supplementary Figure 2. A comparison of mouse and rat PYHIN-encoded protein domains, summarising the results of phylogenetic analyses. Phylogenetic trees show that each rat HIN and pyrin domain is related to a cluster of mouse proteins, indicating gene duplication in mouse. In addition there has been some domain shuffling between genes and probable loss of rat Rhin5 HIN domain. The relationship between mouse and rat proteins is summarised here with colours indicating orthologous pyrin and HIN sequences between mouse and rat and paralogous sequences among mouse genes.
