# Supplementary material for: The mammalian PYHIN gene family: Phylogeny, evolution and expression
Source: BMC Evol Biol. 2012 Aug 7;12:140. doi: 10.1186/1471-2148-12-140 (PMC3458909; doi:10.1186/1471-2148-12-140)
Supplement: Additional file 7 — Table S2. Sequences of primers for real time PCR. [file 1471-2148-12-140-S7.pdf]

Supplementary Table 2. Sequences of primers for realtime PCR analysis

Mouse	Forward	Reverse
Ifi202	GAGAAAAGGAAATGGGAAACC	TCAATGCCACCACTTGTTTG
Ifi203	CTCTCACAAGCAAAGGGGACA	TCCAATTCCATAGCAAATGCAC
Ifi204	TGGAGAACACAGTTTCATCAAGATATC	ACTTGTTTGGGACCATGATGGT
Ifi205	GACTGATCAACTTTTGTGAACGTGTA	GCAGGACTTGCTTCTTGCCTATT
Ifi206	AATACCCAGCAGTTACCAAAATTCC	CCATTTTCTTCAGAAGGTTGTTTAGGT
Ifi207	TTATGGCACCACCAACACCA	TCCTGAGGACCCCTTGTCTTT
Ifi208	TCCAGCAAAGCCACTCAAGG	CAGACTGGGGATTCTGCATTTC
Ifi209	GGAGGTCTACGGACGACTGA	GTGCTTTTTCTTCATCTGGGAT
Aim2	GAATGGGCTGTTTAAAGTCCAGAAG	CCTTCCTCGCACTTTGTTTTGC
Ifi212	CCAGCAGTTCCTCAAACAAGAA	GTTCCTAAAGGTTCTGGAAGCTGA
Ifi213	AATCAGAATCCAGCCCTCCT	GTGAGATCTTAGACGGTGTACCTC
Ifi214	TGGAGTTCTTCTAGCAACACCA	TTTTGGAACCTTGCTGGTAAC
Tbp	GACCTAAAGACCATTGCACTTCGT	GCAGTTGTCCGTGGCTCTCT
Hprt	CAGTCCCAGCGTCGTGATTAG	AAACACTTTTTCCAAATCCTCGG
Cxxc1	CAGACGTCTTTTGGGTCCA	AGACCTCATCAGCTGGCAC
Rpl13A	GAGGTCGGGTGGAAGTACCA	TGCATCTTGGCCTTTTCCTT
