# Supplementary material for: The mammalian PYHIN gene family: Phylogeny, evolution and expression
Source: BMC Evol Biol. 2012 Aug 7;12:140. doi: 10.1186/1471-2148-12-140 (PMC3458909; doi:10.1186/1471-2148-12-140)
Supplement: Additional file 3 — Figure S3. HIN domain alignment. [file 1471-2148-12-140-S3.pdf]

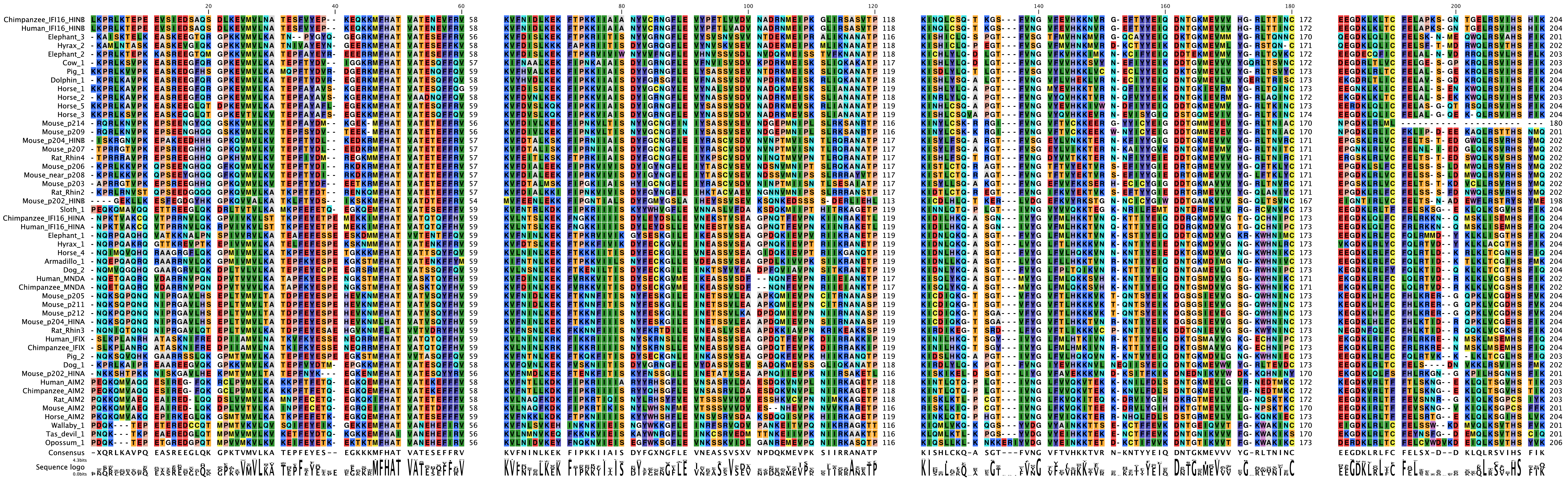

Supplementary Figure 3. ClustalW2 alignment of HIN domains from a range of mammalian species. HIN domain sequences were derived from the two exons encoding the HIN domain. Sequences were obtained from three sources – (i) reference sequences for established genes, (ii) predicted genes in NCBI or Ensembl databases, (iii) de novo exon prediction following identification of sequences homologous to human IFI16 or AIM2 using TBLASTN searches. See Table 1 and Online Resource Table 2 for accession numbers for sequences used. Exon-intron boundaries were well conserved in position and generally aligned well with consensus sequences for splicing. However, the first exon start was unclear for sloth, Tasmanian devil, opossum, and wallaby, and these may instead start from amino acid 3 or 4.
